# Hair embedding after transperineal prostate biopsy: two case reports

**DOI:** 10.1186/s12894-023-01207-8

**Published:** 2023-03-18

**Authors:** Jiaxuan Ni, Ying Ni, Tielong Zhang, Gang Wang, Xuefei Ding, Guangchen Zhou

**Affiliations:** 1 Surgical Research Center, Institute of Urology, Medical School of Southeast University, 210009 Nanjing, Jiangsu China; 2 Department of Urology, Jianhu County People’s Hospital, 224700 Yancheng, China; 3 Department of Urology, Northern Jiangsu People’s Hospital Affiliated to Yangzhou University, 225001 Yangzhou, China

**Keywords:** Hair embedding, Prostate biopsy, Complication, Medical supplies, Adverse event

## Abstract

**Background:**

Transperineal prostate biopsy is gradually becoming the standard methodology for diagnosing prostate cancer because of its high accuracy and low risk of infection, but careful preparation is not always highlighted before a transperineal biopsy.

**Case summary:**

we reported two cases of hair embedding during transurethral resection of the prostate following transperineal puncture biopsy with a Bard MC1820 disposable biopsy needle. Histological examination did not find the hair follicle structure required for hair growth. The hair source was suspected to be percutaneously brought in by needle during the biopsya simulated experiment was used to analyze and reconstruct the process of hair embedding in prostate tissue.

**Conclusion:**

Hair embedding caused by perineal prostate biopsy is a consumable-related adverse event, and skin preparation before a transperineal prostate biopsy is recommended.

## Introduction

Transperineal prostate biopsy is gradually becoming the standard methodology for diagnosing prostate cancer because of its high accuracy and low risk of infection [1]. The complications of invasive puncture include but are not limited to bleeding, infection, urine retention, and vagal reflex, while hair embedding is rare [2, 3]. In this study, we reported two cases of hair embedding as a complication of transperineal prostate biopsy and validated it in a simulation experiment to confirm the process of hair embedding.

### Case presentation

#### Case 1

**Fig. 1:**
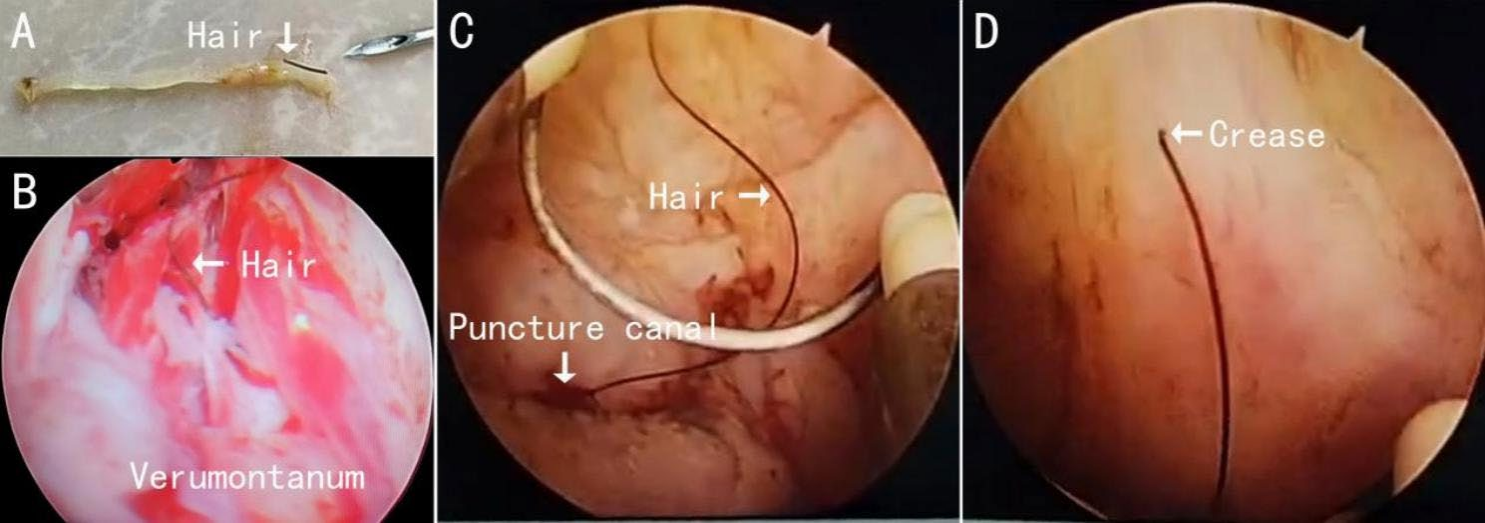
A section of hair was found in the puncture specimen; **B.** Curly hair is found in prostate tissue above verumontanum; **C.** The hair in the puncture channel was found in the process of prostate electrotonic after the puncture, which can distinguish the hair trunk and hair tip; **D.** One end of the hair has a crease.

A 67-year-old male presented with urinary incontinence, dysuria, and hematuria. Anal digital examination revealed that the prostate was approximately 4 cm × 4 cm in size, tough in texture, and free of pain, and the central sulci had vanished. Ultrasound and computerized tomography scans revealed 4 cm × 2.5 cm bladder calculi, blood clots in the bladder, and prostatic hyperplasia. Total prostate-specific antigen (PSA) was over 100 ng/ml in the blood, while free PSA was 12.5 ng/ml. A transperineal prostatic system biopsy was conducted under epidural anesthesia due to budgetary constraints and the patient’s desire to improve life quality, and 12 punctured tissues were retrieved. A hair was discovered on the surface of one specimen (Fig. 1A). Immediately after the puncture, a holmium laser lithotripsy of bladder calculi under a cystoscope was performed, and no trace of hair was found during the surgery. Pathology after paracentesis showed prostate cancer. The patient should have had radical prostatectomy, but because of financial concerns, the patient refused this regimen and only requested transurethral electrostatic resection to relieve symptoms of lower urinary tract obstruction. The patient underwent transurethral electrostatic resection of the prostate one week after the puncture. Curly hair was detected in the prostate tissue above the verumontanum (Fig. 1B). Pathology indicated: prostate adenocarcinoma, Gleason 4 + 4. The patient recovered well after surgery and underwent comprehensive androgen-blocking therapy.

#### Case 2

A 75-year-old man presented with frequent urination, difficulty urinating, and urinary retention. Catheterization was repeated three times in ten days. An anal digital examination revealed that the prostate was 5 × 6 cm in size, medium in texture, with no nodules or discomfort, and the central sulci had vanished. White blood cells in urine: 57/HP. Total PSA was 9.56 ng/ml in the blood, while free PSA was 2.51 ng/ml. Prostatic hyperplasia was discovered using magnetic resonance imaging. Diffusion-weighted imaging revealed a slightly higher signal intensity in the peripheral zone and a slightly thicker bladder wall and right inguinal lymph node. A transperineal prostatic system puncture biopsy was conducted under general anesthesia. The aspiration pathology showed benign prostatic hyperplasia and chronic prostatitis. The patient underwent transurethral electrostatic resection of the prostate two weeks later, during which hair embedding was discovered in the prostatic puncture canal. Figure 1C and 1D show video screenshots. Histopathology following the transurethral electrostatic resection indicated benign hyperplasia with persistent interstitial inflammation; immunohistochemistry: P63 (+), CK (+), P504S (-), AR (+), Ki67 (1%+), PSA (+). The patient healed well postoperatively and was urged to have regular PSA testing.

## Conclusion

The origin of the hair is confusing. The reasons for this phenomenon may be as follows: (1) Hair enters prostate tissue with the needle. Given the patient’s history of perineal prostate puncture surgery and the presence of a wound communicating with the skin in the perineum, it is highly suspected that the needle embedded the residual hair in the prostatic tissue due to insufficient skin preparation; (2) Hair is truly produced in the prostate, such as prostate teratoma. However, extragonadal germ cell tumors are quite rare, accounting for only 1 to 3% of all germ cell tumors[4], while prostate teratoma has rarely been reported on PubMed. According to postoperative histological examination, there was no teratoma component in the resected prostate tissue.

**Fig. 2:**
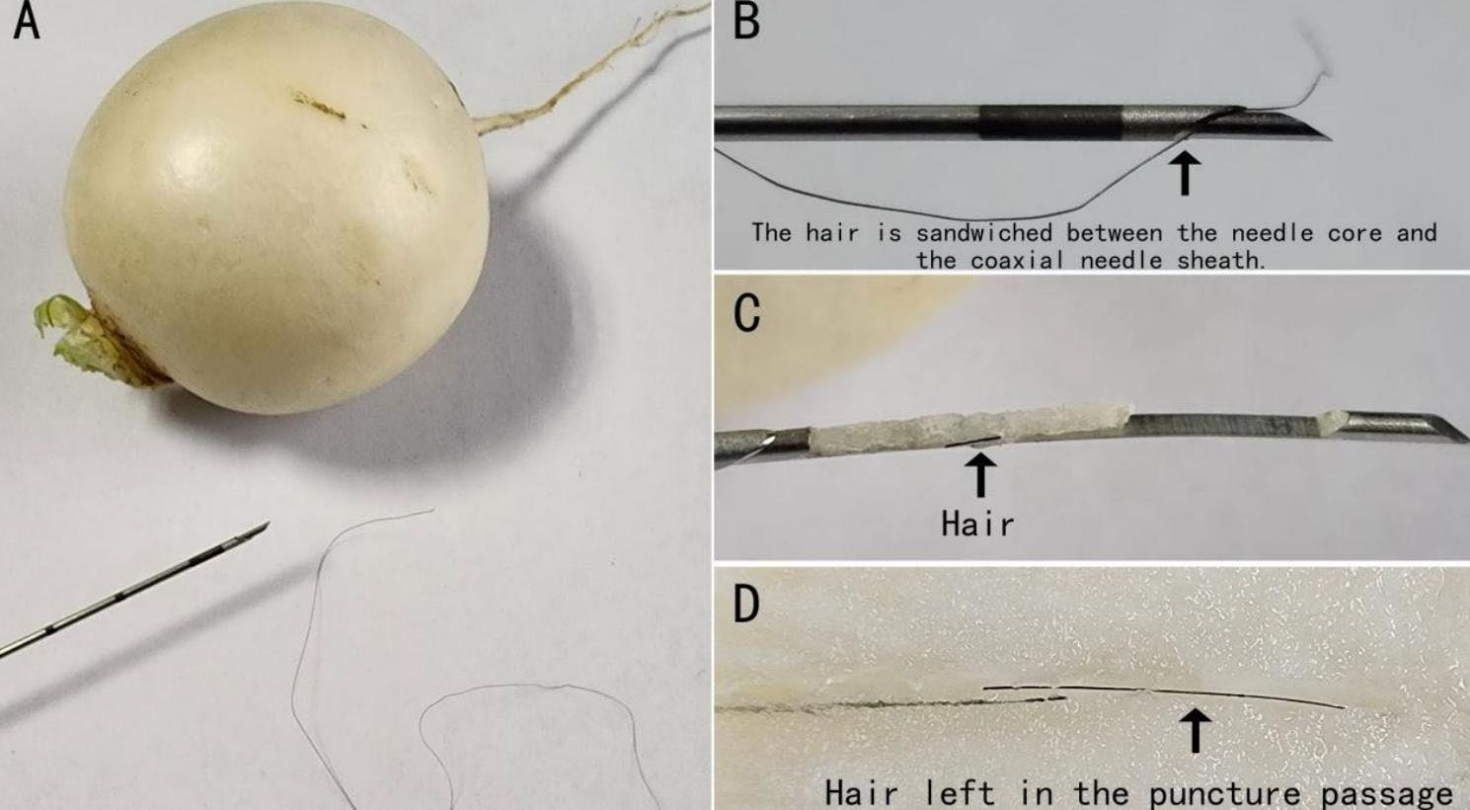
Bard MC1820 disposable biopsy needle, soft hairs, and radish were used for simulation experiment; **B.** The fine hair is easily sandwiched between the needle core and the coaxial needle sheath; **C.** A short hair is seen in the sampling tank; **D.** Retained hair can be seen in the defect area of sampling in the puncture passage.

To verify the possibility of the hair entering the prostate tissue with the needle, a simulation experiment was performed. The materials included Bard MC1820 disposable biopsy needle, soft hairs, a radish, a fruit knife, and a micrometer (Fig. 2A). The object of the simulated puncture was the radish. The outer diameter of the biopsy needle core and the coaxial needle sheath is previously measured with a micrometer. Add a soft hair between the coaxial needle sheath and needle tip before simulated puncture (Fig. 2B). Inspect samples in the sample tank after performing a routine piercing process, and slice the radish along the puncture direction with a fruit knife, then observe where the hair is pushed and kept.

The core diameter of the Bard MC1820 needle is 0.997 mm, while the outer diameter of the coaxial needle sheath is 1.268 mm. The soft hair is easily sandwiched between the needle core and the coaxial needle sheath. After firing the puncture needle, the hair is cut off and visible in the punctured tissue (Fig. 2C), while the hair outside the coaxial needle sheath is pushed and retained in the penetration path (Fig. 2D). A truncated hair was found in the sampling tank, similar to the hair in Fig. 1A.

The prostate gland is the largest accessory gonad in men and is mainly composed of two types of cells, epithelial cells and stromal cells. The prostate was well vascularized and possessed numerous glands and ducts. It has the functions of secreting prostatic fluid and transporting seminal vesicle fluid and sperm[5]. It is well-known that hair follicles are required for hair growth, whereas hair follicles are pouch-like epithelia formed continuously by epidermal cells[6]. This structure generally does not appear in the prostate. Surgeons can confirm and exclude that the hair adheres to the outer sheath and ring of the resectoscope and enters the prostate tissue through the urethra. The implanted hair can be seen in the tissue defect area of the puncture canal in the video of case 2 (screenshot, see Fig. 1C). The visual field can identify the hair shaft’s structure and the hair shaft, and the broken end of the hair shaft exhibits a crease (Fig. 1D). We hypothesize that the hair was wedged between the needle core and the coaxial needle sheath and punctured the deep tissue before being twisted and severed. A simulation study was used to recreate the process of hair embedding in the puncture canal (Fig. 2A, D), and it was proven that the hair was sandwiched between the needle core and the coaxial needle sheath and entered the tissue after the puncture needle broke the skin. After activating the trigger, the hair outside the coaxial needle sheath was forced into the puncture channel and stayed there. The similarity of the break patterns in the experiment’s sample tank and those in Fig. 1A confirms our hypothesis. Curtis et al. reported an instance of hair detected in the material after radical excision of prostate cancer following a transrectal prostate biopsy in 1998, and they suspected that the hair was injected into the prostate by the biopsy needle. Therefore, a transperineal and transrectal prostate puncture may lead to hair insertion, and the root cause is directly related to the biopsy needle used.

Bard MC1820, the author’s most commonly used biopsy needle, is a disposable biopsy needle. The hair entanglement between the needle core and the coaxial needle sheath resulted in the hair embedding into the tissue, which should be attributed to the adverse event related to consumables. If other types of puncture needles are selected or perineal skin preparation is carried out before the puncture, the recurrence of hair embedding can be prevented or reduced.

Skin preparation is one of the essential steps in a transperineal prostate biopsy, and appropriate skin preparation can significantly reduce the risk of surgical site infection. However, given that shaving with a razor in the surgical area may irritate the skin and induce potential infection[7], this institution routinely requires removing the hair from the surgical area with a disposable skin clipper before surgery and then disinfecting the skin with povidone-iodine. Sometimes, to protect privacy, the patient requests the step of hair removal completed by the patient himself or his family. However, because there is no experience or there is a blind area of vision, the hair removal effect may be unsatisfactory, which leaves considerable hidden dangers for imperfect preoperative preparation. Therefore, careful preparation before a transperineal biopsy is necessary to be emphasized. Surgeons should make more specific demands on the degree of skin preparation.

Both patients presented with urinary obstruction as a cause and underwent timely further surgery. If case 1 did choose radical prostatectomy because of not being concerned about economic factors, residual hair follicles may even appear in the pathology report. But due to the increasing popularity of prostate cancer screening techniques, more and more people will undergo prostate biopsies without symptoms. If the patient’s hair is embedded during the puncture without subsequent treatment, it may cause adverse consequences such as prostatic inflammation and infection. Hair granuloma will be found in the pathological section if the patient with hair embedded due to puncture is diagnosed with prostate cancer and has undergone radical prostate cancer surgery [8–11]. The main advantage of transperineal prostate biopsy over the transrectal route is the lower infection and bleeding rates, but this advantage may no longer exist once hair impaction occurs. One would therefore say that the lack of precision in preparing perineal skin could lead to a higher risk of infection compared to a transrectal biopsy. However, it is still unknown whether the foreign body reaction caused by the hair in the prostate tissue of patients who have not been diagnosed with prostate cancer or have not undergone radical prostatectomy will lead to persistent PSA abnormalities, perineal pain, prostatitis syndrome, medical image changes, etc.

## Data Availability

All data generated or analyzed during this study are included in this published article.

## Abbreviations

PSA: prostate-specific antigen
